# Cardiovascular Events in Patients With Acute Myeloid Leukemia Treated With Venetoclax: A Multicenter Cohort Study

**DOI:** 10.1016/j.jacadv.2026.102821

**Published:** 2026-06-17

**Authors:** Sabin Filimon, Azin Ghamari, Azin Vakilpour, Ruchi Patel, Rohan Achar, Nausheen Akhter, Rory M. Shallis, Jeanne M. DeCara, Jessica Altman, Amanda Smith, Andrew Matthews, Catherine Lai, Andrew M. Brunner, Kyle A. Farina, Hannah K. Gilman, Tomas G. Neilan, Deep Upadhyay, Jesse Chittams, Francisca Anazco, Anand A. Patel, Douglas Tremblay, Marielle Scherrer-Crosbie

**Affiliations:** a Division of Cardiovascular Diseases, Department of Medicine, Hospital of the University of Pennsylvania, Philadelphia, Pennsylvania, USA; b Thalheimer Center for Cardio-Oncology, Division of Cardiology and Abramson Cancer Center, Department of Medicine, Perelman School of Medicine at the University of Pennsylvania, Philadelphia, Pennsylvania, USA; c Cardio-Oncology Program, Division of Cardiology, Massachusetts General Hospital, Boston, Massachusetts, USA; d Division of Cardiology, Feinberg School of Medicine, Northwestern University, Chicago, Illinois, USA; e Division of Medical Oncology and Hematology, Department of Internal Medicine, Yale School of Medicine and Yale Cancer Center, New Haven, Connecticut, USA; f Division of Cardiology, Department of Medicine, University of Chicago, Chicago, Illinois, USA; g Division of Hematology-Oncology, Feinberg School of Medicine, Northwestern University, Chicago, Illinois, USA; h Division of Hematology-Oncology, Department of Medicine, Hospital of the University of Pennsylvania, Philadelphia, Pennsylvania, USA; i Leukemia Program, Massachusetts General Hospital, Harvard Medical School, Boston, Massachusetts, USA; j Tisch Cancer Institute, Icahn School of Medicine at Mount Sinai, New York City, New York, USA; k Feinberg School of Medicine, Northwestern University, Chicago, Illinois, USA; l Department of Biobehavioral Health Sciences, School of Nursing, University of Pennsylvania, Philadelphia, Pennsylvania, USA; m Division of Hematology-Oncology, Department of Medicine, University of Chicago, Chicago, Illinois, USA

**Keywords:** cardiotoxicity, hypomethylating agents, venetoclax

## Abstract

**Background:**

Patients with acute myeloid leukemia (AML) treated with hypomethylating agents and venetoclax (HMA-VEN) may be at risk for cardiovascular complications. The incidence, associations, and prognostic implications of these events remain poorly defined.

**Objectives:**

The purpose of this study was to evaluate the incidence, risk factors, and prognostic significance of major adverse cardiovascular events (MACE) in AML patients treated with HMA-VEN and to identify clinical and genetic predictors.

**Methods:**

We conducted a multicenter retrospective cohort study across 6 U.S. health care systems between January 2017 and July 2024. The study included 1,012 adults (mean age 69 ± 12.5 years; 57% male) with newly diagnosed or relapsed/refractory AML treated with HMA-VEN. The primary outcome was MACE, defined as atrial fibrillation, heart failure, left ventricular ejection fraction reduction, stroke/transient ischemic attack, acute coronary syndrome, angina, ventricular tachycardia/fibrillation, myocarditis, or cardiovascular death. The secondary outcome was noncardiac mortality. Variables associated with MACE and mortality were analyzed using Fine-Gray competing risk and Cox proportional hazards models.

**Results:**

MACE occurred in 20% of patients (n = 200), with a median time to first MACE of 120 days and a 12-month cumulative incidence of 17%. Atrial fibrillation (6%), stroke/transient ischemic attack (5%), left ventricular ejection fraction reduction (5%), and heart failure (4%) were most frequent. Diabetes independently predicted MACE (subdistribution HR: 1.4; 95% CI: 1.03-1.89; *P* = 0.031). In the matched cohort, MACE was associated with increased mortality (73% vs 56%; HR: 1.96; 95% CI: 1.59-2.42; *P* < 0.001).

**Conclusions:**

MACE are frequent and occur early in patients with AML treated with HMA-VEN, particularly in patients with diabetes, and are associated with lower survival.

Venetoclax (VEN), a selective B-cell lymphoma-2 inhibitor, is an essential and increasingly used therapy in combination with hypomethylating agents (HMAs) for patients with acute myeloid leukemia (AML) unfit for intensive chemotherapy.^1^^,^^2^

Patients with AML are intrinsically vulnerable to cardiovascular (CV) complications due to advanced age, a high prevalence of traditional CV risk factors, systemic inflammation, and metabolic stress related to both disease and treatment.^3^ CV events have been reported with HMA-based regimens, including heart failure (HF), arrhythmias, and ischemic events.^4^, ^5^, ^6^, ^7^, ^8^, ^9^, ^10^ The addition of VEN may further increase CV risk, supported by preclinical evidence of VEN-associated myocardial injury mediated by oxidative stress and apoptosis, as well as emerging clinical reports describing early CV events during HMA-VEN therapy.^11^ However, available clinical data remain limited to small, single-center studies with heterogeneous definitions of events.^12^^,^^13^

Accordingly, we conducted a large multicenter retrospective cohort study across 6 U.S. health care systems to characterize major adverse cardiovascular events (MACE) in patients with AML treated with HMA-VEN. Using competing-risk models and time-dependent propensity score matching, we sought to define the incidence and predictors of CV events and to evaluate their association with subsequent mortality. These data aim to inform CV risk stratification and multidisciplinary management in patients receiving HMA-VEN therapy. The intent of this study was not to establish causality or attribute CV events solely to HMA-VEN, but rather to characterize the incidence, phenotypes, predictors, and prognostic implications of CV events within this treated population. Accordingly, our analytic framework was designed to focus on cumulative incidence and associations rather than comparisons across treatment groups.

## Methods

### Population

The medical records of all consecutive adult patients (≥18 years of age) with newly diagnosed or remitting/relapsing AML treated with HMA-VEN between January 2017 and July 2024 were collected from 6 independent health care systems (Supplemental Table 1). The present multicenter cohort includes patients previously reported in a single-center study from the University of Pennsylvania.^13^ Specifically, 103 patients from the prior publication were included in the current analysis, along with an additional 92 Penn patients subsequently identified, for a total of 195 patients contributed by University of Pennsylvania. There was no overlap with patients from other participating institutions.

### Data collection

After approval by the local Institutional Review Board at each site, clinical data were manually collected. Medical charts were reviewed individually to identify baseline characteristics and major adverse cardiovascular events (MACE). The entry date was defined as the date of VEN initiation.

### Baseline characteristics

Baseline characteristics were assessed at the encounter closest in time before the initiation of HMA-VEN therapy. CV disease was defined as the presence of pre-existing coronary artery disease (CAD), chronic HF, atrial fibrillation (AF), or a history of stroke. CV risk factors were defined as hypertension, dyslipidemia, diabetes mellitus, obesity, and tobacco use. Data were collected on common AML mutations associated with CV complications and mutations associated with clonal hematopoiesis of indeterminate potential. Baseline left ventricular ejection fraction (LVEF) assessment by transthoracic echocardiography at the time of AML diagnosis was available in 83% of patients.

### Major adverse cardiovascular events

MACE was defined as a composite outcome, occurring throughout the follow-up period (throughout July 2024), including de novo symptomatic HF, decrease in LVEF, AF, ventricular fibrillation/tachycardia, angina, acute coronary syndrome (ACS), arterial thromboembolism, stroke/transient ischemic attack (TIA), myocarditis, and death attributed to cardiac causes. MACE were defined using the American College of Cardiology/American Heart Association outcome definitions for clinical trials and were graded according to the Common Terminology Criteria for Adverse Events (CTCAE) Version 5.0.^14^^,^^15^

De novo LVEF reduction was defined according to the International Cardio-Oncology Society consensus statement^16^ as a new LVEF reduction by ≥10% from baseline to a final value of <50%. De novo cardiac arrhythmias were identified on electrocardiograms. Chronic, stable cardiac disease, such as in a patient with known HF who tolerated treatment without an LVEF decrease or symptomatic exacerbation, was not classified as a cardiac event. Patients with sepsis or critical illness at the time of MACE were excluded. MACE of the same category was counted only once per patient. In case of uncertainty, CV events were adjudicated by 2 independent cardiologists (S.F. and N.A.), with a third cardiologist (M.S.-C.) consulted in case of disagreement.

### Noncardiac mortality

Noncardiac mortality was documented and included deaths resulting from septic shock, multiple organ failure, or cancer progression.

### Statistical analysis

Categorical data are presented as counts and percentages, while continuous data are reported as mean ± SD or median (IQR: Q1–Q3). Median follow-up time was estimated using the reverse Kaplan-Meier method. Time to death was analyzed using the Kaplan-Meier method, with patients alive at last follow-up censored. Time to first MACE was used as the elapsed time in days for patients with multiple MACE. Differences between patients with and without MACE were determined using the chi-square test for categorical variables and the independent samples *t*-test or Mann-Whitney *U* test for continuous variables. Normality was assessed using the Kolmogorov-Smirnov test. The cumulative incidence function was used to estimate the incidence of MACE, with noncardiac death considered as a competing event. Univariable Fine and Gray’s subdistribution hazard regression model was used to determine the association between demographic and clinical variables and MACE. Multivariable Fine-Gray subdistribution hazard models were constructed to estimate the independent association between covariates and MACE. Covariates were selected based on a combination of clinical relevance and variables demonstrating a potential association in univariable analyses (*P* < 0.10) to improve model specification, while minimizing multicollinearity. The results of Fine and Gray’s models were expressed as subdistribution HRs (sHRs) with 95% CIs. All statistical analyses were performed using SPSS (IBM SPSS version 26) or R version 4.3.0. A *P* value of <0.05 was considered statistically significant, and all analyses were considered exploratory.

To evaluate the impact of MACE on subsequent mortality, we conducted a subgroup analysis using time-dependent propensity score matching. Patients who experienced a MACE event were matched with up to 6 patients who did not experience MACE but survived at least as long as the index time of the corresponding case. The index date for time-to-event analysis was defined as the date of MACE for cases and the corresponding matched time for controls. The primary outcome was all-cause mortality. Time-to-event was calculated from the index date to the date of death or last follow-up. Propensity score matching was performed using the variable ratio (kmin = 1, kmax = 6) matching method with a caliper of 0.68 in SAS version 9.4. This approach identifies the optimal set of matches by minimizing the total absolute difference in propensity scores across all matched pairs, rather than using a greedy or nearest-neighbor algorithm. The method yields an average matching ratio approximating the midpoint of kmin and kmax. Propensity scores were estimated without inclusion of additional covariates. Univariable and multivariable Cox proportional hazards models were used to estimate HRs and 95% CIs, with robust standard errors clustered by matched pairs to account for the matched cohort design.

## Results

### Baseline characteristics

Between January 2017 and July 2024, 1,012 patients with AML who received HMA-VEN as frontline or remitting/relapsing AML treatment were identified (69 ± 12.5 years old, 57% male, 79% White, 10% Black, 1% Hispanic) (Table 1). Hypertension was present in 60%, dyslipidemia in 49%, and diabetes mellitus in 22%. Forty-nine patients had secondary AML, 35% had a history of prior malignancy, and 46% received previous cancer therapies. Prior anthracycline exposure was 22% and stem cell transplant (SCT) 29%.

**Table 1:** Baseline Characteristics of Patients Treated With HMA-VEN

	Total (N = 1,012)	MACE (n = 200)	No MACE (n = 812)	*P* Value
Baseline demographic and cardiovascular characteristics				
Age, y	69 ± 12.5	70 ± 12	69 ± 13	0.284
Male	577 (57)	121 (60)	456 (56)	0.266
Race				0.043
White	805 (79)	156 (78)	649 (80)	
Black	99 (10)	28 (14)	71 (9)	
Other/unknown	108 (11)	16 (8)	92 (11)	
BMI (kg/m^2^)	27 ± 6	27 ± 6	27 ± 6	0.781
Obesity	256 (25)	54 (27)	202 (25)	0.536
Active smoker	63 (6)	7 (3.5)	56 (7)	0.075
Past smoker	416 (41)	85 (43)	331 (41)	0.655
Diabetes	277 (22)	55 (27)	172 (21)	0.055
Dyslipidemia	493 (49)	103 (52)	390 (48)	0.379
Hypertension	612 (60)	125 (62)	487 (60)	0.513
History of coronary artery disease	212 (21)	56 (28)	156 (19)	0.006
History of stroke/TIA	76 (7)	18 (9)	58 (7)	0.372
Chronic kidney disease	240 (24)	46 (23)	194 (24)	0.791
History of atrial fibrillation	216 (21)	40 (20)	176 (22)	0.605
History of heart failure	130 (13)	27 (14)	103 (13)	0.758
Chronic obstructive pulmonary disease	120 (12)	27 (13)	93 (11)	0.423
Screening TTE at diagnosis	836 (83)	183 (92)	653 (80)	<0.001
MRI or MUGA at diagnosis	12 (1)	6 (3)	6 (1)	0.008
LVEF at diagnosis (n = 836)	61 ± 8	60 ± 9	61 ± 8	0.508
Cancer characteristics				
Previous history of cancer	359 (35)	66 (33)	293 (36)	0.414
De novo AML	511 (51)	100 (50)	411 (51)	0.851
Secondary AML^a^	501 (49)	100 (50)	401 (49)	0.876
Baseline treatment and medication				
Previous cancer therapies	461 (46)	76 (38)	385 (48)	0.016
Past anthracyclines	223 (22)	36 (18)	187 (23)	0.912
Total prior cumulative dose of anthracycline (mg/m^2^)^b^	162 ± 83	166 ± 73	161 ± 85	0.782
Previous SCT	288 (29)	49 (24)	239 (30)	0.166
SCT before venetoclax	149 (15)	24 (12)	125 (15)	0.225
SCT after venetoclax	156 (15)	32 (16)	124 (15)	0.798
SCT both before and after venetoclax	31 (3)	6 (3)	25 (3)	0.954
Previous thoracic radiation	83 (8)	10 (5)	73 (9)	0.065
Number of venetoclax cycles received	3 [2-6]	3 [2-6]	3 [2-6]	0.138
Azacitidine	680 (67)	150 (75)	530 (65)	0.009
Decitabine	359 (35)	59 (30)	300 (37)	0.049
Venetoclax interruption	530 (52)	106 (53)	424 (52)	0.843
Time from AML diagnosis and initiation of venetoclax-HMA (d)	14 [6-56]	13 [6-35]	14 [6-63]	0.420
ACE/ARB	262 (26)	61 (31)	201 (25)	0.097
ARNI	18 (2)	6 (3)	12 (1)	0.145
MRA	36 (4)	14 (7)	22 (3)	0.003
Beta-blockers	356 (35)	94 (47)	262 (32)	<0.001
SGLT2i	26 (3)	4 (2)	22 (3)	0.570
Statins	405 (40)	88 (45)	317 (39)	0.200
Aspirin	168 (17)	51 (26)	117 (14)	<0.001
Genomics				
JAK2	69 (7)	15 (7)	54 (7)	0.656
DNMT3A	169 (17)	33 (17)	136 (17)	0.956
TET2	190 (19)	39 (20)	151 (19)	0.745
ASXL1	165 (16)	33 (17)	132 (16)	0.911
TP53	281 (28)	50 (25)	231 (28)	0.358
IDH1	80 (8)	18 (9)	62 (8)	0.518
IDH2	107 (11)	25 (12)	82 (10)	0.319
FLT3	184 (18)	40 (20)	144 (18)	0.452
NPM1	136 (13)	29 (15)	107 (13)	0.618
NRAS	102 (10)	21 (10)	81 (10)	0.808
KRAS	54 (5)	9 (4)	45 (6)	0.567
RUNX1	161 (16)	36 (18)	125 (15)	0.366
SF3B1	48 (5)	10 (5)	38 (5)	0.837
SRSF2	123 (12)	29 (15)	94 (11)	0.246
IDH1/IDH2	177 (17)	42 (21)	135 (17)	0.145
DNMT3A/TET2/ASXL1	408 (40)	78 (39)	330 (41)	0.672
ECOG performance status at the venetoclax initiation				0.255
ECOG 0	200 (20)	36 (18)	164 (20)	
ECOG 1	452 (45)	90 (45)	362 (45)	
ECOG 2	227 (23)	39 (19)	188 (23)	
ECOG 3	59 (6)	14 (7)	45 (6)	
ECOG 4	7 (1)	2 (1)	5 (0.6)	

Values are median [IQR], n (%), or mean ± SD.

ACE = angiotensin-converting enzyme; AML = acute myeloid leukemia; ARB = angiotensin receptor blocker; ARNI = angiotensin receptor-neprilysin inhibitor; BMI = body mass index; ECOG = Eastern Cooperative Oncology Group; HMA = hypomethylating agent; LVEF = left ventricular ejection fraction; MRA = mineralocorticoid receptor antagonist; MRI = magnetic resonance imaging; MUGA = multigated acquisition scan; SCT = stem cell transplant; SGLT2i = sodium glucose co-transporter 2 inhibitor; TIA = transient ischemic attack; TTE = transthoracic echocardiography; VEN = venetoclax.

a Post-myelodysplastic syndrome = 258 (51.5%), therapy related = 133 (26.5%), secondary to myeloproliferative neoplasm = 110 (22%).

b Data in 172 patients.

### Major adverse cardiovascular events

A total of 200 patients (20%) experienced MACE, with a total of 282 CV events over a median follow-up period of 280 days (IQR: 98-580; range 2-2,615 days) (Table 2). The most frequent events were de novo AF (6%, n = 62), stroke/TIA (5%, n = 53), de novo LVEF reduction (5%, n = 52), and new-onset HF (4%, n = 38). The characteristics of the individual CV events are detailed in Supplemental Table 2.

**Table 2:** Events in 1,012 Patients Treated With HMA-VEN

Event	n (%)	Time to Event^a^, Median Days [IQR]
MACE, n^b^	282	120 [19-521]
De novo AF	62 (6)	124 [16-489]
Stroke/TIA	53 (5)	122 [34-362]
De novo decreased LVEF	52 (5)	102 [33-255]
De novo HF	38 (4)	128 [27-474]
Angina	26 (3)	212 [107-538]
ACS	23 (2)	121 [62-356]
VT/VF	19 (2)	160 [34-574]
Cardiovascular death	9 (0.9)	156 [31-187]
Myocarditis	8 (0.8)	119 [65-300]
Arterial thromboembolism	1 (0.1)	34

**Death**	**n (%)**	**Time to Death, Median Days [IQR]**

Total cohort	676 (67)	217 [78-415]
MACE	149 (74)	277 [104-528]
No MACE	527 (65)	199 [73-393]

ACS = acute coronary syndrome; AF = atrial fibrillation; HF = heart failure; MACE = major adverse cardiovascular events; VT/VF = ventricular tachycardia/ventricular fibrillation; other abbreviations as in Table 1.

a In patients with multiple MACE, time to earliest MACE was selected.

b 40 patients had 2 MACE, 9 patients had 3 MACE, 3 patients had 4 MACE, 1 patient had 5 MACE, and 1 patient had 8 MACE.

Among the 200 patients with a CV event, 54 (27%) had more than 1 event; 40 patients had 2 events, 9 patients had 3, 3 patients had 4, 1 patient had 5, and 1 patient had 8. In patients with multiple events, LVEF reduction (26%), de novo AF (17%), ACS (17%), and de novo HF (11%) were the most frequent initial events. The median time to the first CV event was 120 days (IQR: 19–521).

**Figure 1:**
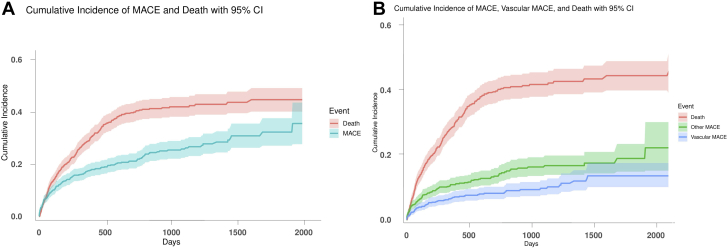
**Cumulative Incidence of MACE and Noncardiovascular Mortality in Acute Myeloid Leukemia** Kaplan-Meier cumulative incidence curves illustrate MACE and noncardiovascular mortality in patients with acute myeloid leukemia treated with hypomethylating agents and venetoclax. Panel (A) shows overall MACE (blue) with death (black) treated as a competing risk. Panel (B) depicts vascular MACE (blue), other MACE (green), and death (red). Vascular MACE include stroke/transient ischemic attack, acute coronary syndrome, angina, and arterial thromboembolism, whereas other MACE include atrial fibrillation, heart failure, left ventricular ejection fraction reduction, myocarditis, and ventricular arrhythmias. Shaded areas represent 95% CIs.

The cumulative incidence of MACE at 12 months was 17% (Figure 1). A cumulative incidence analysis stratified by type of MACE, distinguishing between vascular MACE (stroke/TIA, ACS, angina, and arterial thromboembolism) and other MACE revealed that the cumulative incidence of vascular MACE was 7.5% at 12 months (Figure 1). The presence of MACE led to the interruption of VEN in 106 of the 200 patients (53%).

Thirty-eight patients developed de novo HF at a median time of 128 days (IQR: 27-474 days). The baseline LVEF was 59% ± 10%. Thirteen patients (34%) were diagnosed with HF with preserved ejection fraction (HFpEF). In the 25 remaining patients, LVEF decreased from 57% ± 11% at baseline to 34 ± 10% at the time of the event. N-terminal pro–B-type natriuretic peptide (NT-proBNP) levels rose from a baseline value of 896 pg/mL (IQR: 94-3,100 pg/mL) to 3,562 pg/mL (IQR: 1,144-16,905) at the time point closest to diagnosis. Based on CTCAE v5.0 criteria, HF was classified as Grade 2 in 45% of cases and Grades 3 and 4 in 55%. Takotsubo cardiomyopathy was identified in 9 patients (24% of all patients with HF), and valvular diseases or arrhythmias were thought to contribute in 4 patients (10% of all patients with HF).

Fifty-two patients had LVEF reduction at a median time of 102 days after HMA-VEN initiation, with a mean reduction of 19% ± 9%. Of the 50 patients who had a follow-up echo after the initial decrease, 8 (15%) achieved recovery (LVEF>50%). NT-proBNP levels increased from a median value of 479 pg/mL (IQR: 99-4,444 pg/mL) to 6,940 pg/mL (IQR: 1,316-18,121 pg/mL) at the time point closest to the observed decrease in LVEF.

Twenty-three patients developed ACS at a median time of 121 days. Coronary angioplasty/stent was performed in 8 of these 23 patients (35%). Twenty-six patients developed angina at a median time of 212 days.

Myocarditis was detected in 8 patients (0.8%). The diagnosis was confirmed by cardiac magnetic resonance imaging in 6 patients, including 1 case further confirmed by biopsy. At the time of diagnosis, patients had a mean LVEF of 50% ± 20% and elevated NT-proBNP levels (5,696 pg/mL [IQR: 1,346-12,188 pg/mL]).

**Figure 2:**
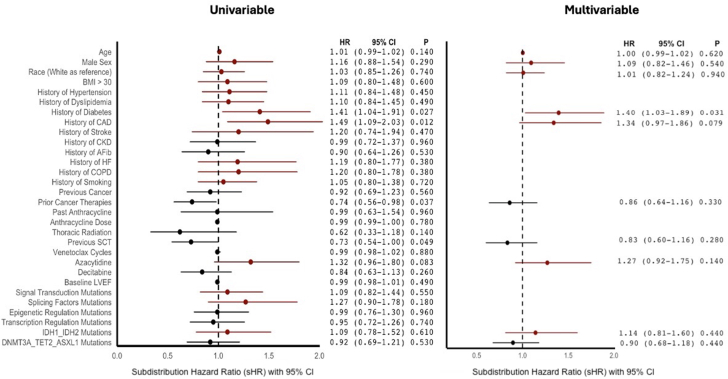
**Competing Risk Analysis of Variables Associated With MACE** Forest plots display subdistribution HRs with 95% CIs for variables associated with MACE using Fine-Gray competing risk models. Univariable (left) and multivariable (right) analyses are shown. In univariable analysis, diabetes and prior coronary artery disease were associated with increased subdistribution hazard of the composite outcome of MACE. In multivariable analysis, diabetes remained independently associated with MACE, whereas other variables were not statistically significant after adjustment. Estimates are presented as subdistribution HRs with corresponding 95% CIs, and the dashed vertical line indicates no association (subdistribution HR = 1). BMI = body mass index; CAD = coronary artery disease; CKD = chronic kidney disease; COPD = chronic obstructive pulmonary disease; HF = heart failure; IDH = isocitrate dehydrogenase; LVEF = left ventricular ejection fraction; SCT = stem cell transplant.

In a univariable Fine-Gray competing risk analysis, diabetes (sHR: 1.41; 95% CI: 1.04-1.91; *P* = 0.027) and a history of CAD (sHR: 1.49; 95% CI: 1.09-2.03; *P* = 0.012) were associated with an increased subdistribution hazard of the composite outcome of MACE. Diabetes remained associated with MACE (sHR: 1.4; 95% CI: 1.03-1.89; *P* = 0.031) in a multivariable analysis including age, sex, race, CAD, prior cancer therapies/SCT, and genomic mutations (Figure 2). Black race and Hispanic ethnicity were associated with a higher incidence of HF/LVEF events in a univariable analysis (sHR: 1.69; 95% CI: 1.05-2.72; *P* = 0.031) (Supplemental Table 3), while past anthracycline exposure remained a significant predictor in the multivariable model (sHR: 2.08; 95% CI: 1.04-4.14; *P* = 0.038) (Supplemental Table 4). For vascular events, both obesity and diabetes were associated with an increased subdistribution hazard in univariable analysis; however, only diabetes remained significant in multivariable analysis (sHR: 1.80; 95% CI: 1.16-2.80; *P* = 0.008) (Supplemental Tables 5 and 6). Prior anthracycline exposure was specifically examined in prespecified Fine-Gray competing risk analyses. Prior anthracycline exposure was not associated with the composite outcome of HF or LVEF reduction (sHR: 1.10; 95% CI: 0.70-1.72; *P* = 0.69) (Supplemental Table 3) nor with vascular MACE (Supplemental Tables 5 and 6).

Overall mortality in the cohort was 67% (n = 676), with a median time to death of 217 days (IQR: 78-415). CV mortality was 0.9% (n = 9) and occurred at a median of 156 days (IQR: 31-187). Among patients with MACE, mortality was 74% (n = 149), whereas it was 65% (n = 527) in patients with no MACE (Table 2).

### Time-dependent propensity score matching analysis

A total of 645 patients were included: 200 who experienced MACE and 445 matched controls without MACE who survived long enough to be eligible for comparison. The time-dependent matching procedure was designed to align patients based on time at risk, whereby patients with MACE were matched to patients without MACE who were alive and under follow-up at the corresponding time point. Matching was performed solely on follow-up time, and no demographic or clinical covariates were included in the matching process. Baseline demographic and clinical variables, including age and CV comorbidities, were not incorporated into the matching algorithm but were instead adjusted for in subsequent multivariable Cox proportional hazards models to avoid overadjustment and collinearity. In a univariable Cox proportional hazards analysis, MACE was significantly associated with death (HR: 1.92; 95% CI: 1.56-2.36; *P* < 0.001). This association remained highly significant (HR: 1.96; 95% CI: 1.59-2.42; *P* < 0.001) in a multivariable analysis including age, sex, hypertension, diabetes, chronic kidney disease, history of CAD, history of HF, and prior cancer therapies (Supplemental Table 7). Other independent variables associated with mortality included older age (HR: 1.01; 95% CI: 1.00-1.02; *P* = 0.006), history of CAD (HR 1.35, 95% CI: 1.05-1.74; *P* = 0.020), and treatment with prior cancer therapy (HR: 1.25; 95% CI: 1.02-1.54; *P* = 0.035).

## Discussion

In this large multicenter retrospective study, 20% of patients with AML treated with HMA-VEN experienced a CV event, with a median time to first event of 120 days and a 12-month cumulative incidence of 17%. The most common events were de novo AF, LVEF reduction, and HF, along with stroke/TIA. A history of diabetes or CAD was associated with an increased risk of CV events; diabetes remained significant after accounting for age, sex, race, CAD, prior cancer therapies/SCT, and genomic mutations. Black race, Hispanic ethnicity, and prior anthracycline therapy were associated with a higher incidence of HF/LVEF in univariable analysis. The overall mortality in the cohort was high (67%), and the occurrence of CV events was associated with increased mortality. The key findings of this study are summarized in the Central Illustration.

**Central Illustration:**
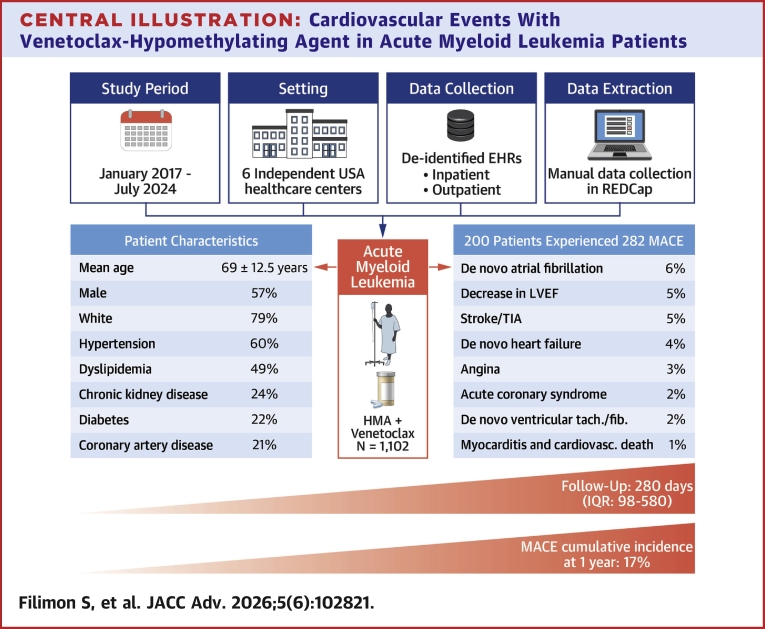
**Cardiovascular Events With Venetoclax-H**ypomethylating Agent **in Acute Myeloid Leukemia Patients** This central illustration summarizes the incidence, timing, predictors, and outcomes of major adverse cardiovascular events (MACE) in patients with acute myeloid leukemia treated with hypomethylating agents and venetoclax. In a multicenter cohort of 1,012 patients, 20% experienced MACE, most commonly atrial fibrillation, heart failure, and **left** ventricular ejection fraction decline, with a median onset of 120 days. Diabetes and prior coronary artery disease emerged as key predictors of cardiovascular risk. MACE were associated with increased mortality and frequently led to treatment interruption. These findings highlight the incidence of cardiovascular events among acute myeloid leukemia patients treated with hypomethylating agent–venetoclax therapy and underscore the importance of baseline risk stratification and close cardiovascular monitoring in this high-risk population. EHRs = Electronic Health Records; HMA = hypomethylating agent; TIA = transient ischemic attack; other abbreviation as in Figure 2.

Two other retrospective studies from a single institution reported CV events in patients with AML treated with HMA-VEN.^12^^,^^17^ In an earlier study of 170 patients, 20% of patients experienced CV events,^12^ a proportion similar to that found in our study. Although the second study from the same single institution reported a lower rate of CV events (7.6%), only LVEF decrease, non–ST-segment elevation myocardial infarction, and pericarditis/pericardial effusion were reported.^17^ Pericardial events were not included in the present study, as this finding is frequent in patients with cancer and is considered nonspecific.^18^ The broader event definition and multicenter design of the present report likely captured a more comprehensive characterization of the patients and of the CV events. Despite differences, all studies highlight a high burden of early CV events in AML patients treated with HMA-VEN, underscoring the need for close CV monitoring.

The burden of CV events in patients with AML treated with HMA-VEN is further highlighted by the substantial proportion of patients who experienced multiple CV events. Over one-quarter (27%) of patients with the composite outcome of MACE had more than one event, with some experiencing up to 8 distinct cardiac complications. The most common first events were a reduction in LVEF, de novo AF, and ACS.

In the present study, de novo AF was the most frequent CV event, affecting 6% of the total cohort. This rate is markedly higher than that reported in an age-matched general population (1.7% in women and 3% in men).^19^ AF has been reported in 2.4% to 14.6% of cancer patients, with prevalence varying by cancer type and patient age.^20^^,^^21^ In AML patients receiving anthracyclines, Onoue et al reported AF in 6.3% of patients.^22^ In a matched cohort from our institution comparing AML patients receiving VEN vs anthracyclines, de novo AF occurred in 12% vs 6% of patients, respectively.^13^ Notably, the VEN-treated cohort in that study was slightly older than ours (median age 71 vs 69 years), which may have contributed to the higher AF incidence observed. The predominance of AF may reflect the advanced age and high burden of baseline CV comorbidities, including hypertension and structural heart disease, in addition to the proarrhythmic and inflammatory stressors associated with both AML and its treatments.

Thirty-eight patients developed de novo HF, presenting with significant heterogeneity in etiology and severity. Among patients with decreased LVEF, only 15% recovered the LVEF ≥50%, indicating limited reversibility. Most cases were classified as CTCAE v5.0 Grade 3 or 4 (55%), underscoring the severity of these events. Takotsubo cardiomyopathy was diagnosed in 24% of patients with HF, and 0.1% of the total cohort; the prevalence of Takotsubo cardiomyopathy is thought to be 0.02% of all hospitalized patients.^23^ The present findings highlight the importance of considering Takotsubo as an etiology of HF in patients with AML treated with HMA-VEN. A retrospective study reported that in patients with cancer, Takotsubo cardiomyopathy was most frequently associated with lymphoproliferative neoplasms.^24^ Notably, when this condition occurs in a patient with malignancy, it is associated with worse outcomes compared to the general population.^25^ No clear etiology was identified in 13 (34%) patients, which may reflect the multifactorial nature of HF in this population. HFpEF accounted for 34% of cases. While not explicitly categorized in CTCAE v5.0, HFpEF is common in patients with cancer due to emerging evidence suggesting it is prevalent in older oncology cohorts with comorbidities such as hypertension, CAD, and chronic kidney disease.^26^ Prior cancer therapies, including anthracyclines, vascular endothelial growth factor inhibitors, and SCT, may contribute to HFpEF via microvascular dysfunction, inflammation, and increased arterial stiffness.^26^^,^^27^

The high rate of MACE in patients with AML treated with HMA-VEN may be due to several factors. The high prevalence of hypertension (60%), diabetes (22%), and CAD (21%) in the present cohort exceeds that observed in Framingham participants of comparable age, where rates of hypertension and diabetes are typically around 40% and 10 to 15%, respectively.^28^^,^^29^ Notably, a substantial proportion of patients had pre-existing cardiac conditions, including AF and HF. This elevated baseline CV risk reflects the cumulative burden of common risk factors for CV disease and cancer, including age, CV risk factors, clonal hematopoiesis of indeterminate potential mutations.

VEN could also be cardiotoxic through several mechanisms. Preclinical studies have demonstrated that VEN treatment can induce myocardial injury by promoting oxidative stress, inflammation, and apoptosis.^11^ Prior work from our group showed that patients treated with VEN experienced rates of CV events comparable to, or even exceeding, those observed with anthracyclines, particularly during the early phase of treatment.^13^ A recent randomized trial identified AF in 5% of patients treated with azacitidine-VEN compared to 1% in the azacitidine-only group.^1^ These findings raise concerns about the potential cardiotoxicity of VEN, particularly when combined with HMAs.

Separating the CV effects of prior anthracycline exposure from those of VEN is challenging in observational AML studies. In prespecified competing risk analyses, prior anthracycline exposure was not independently associated with HF, LVEF reduction, or vascular MACE. These findings suggest that the observed CV events reflect the combined influence of baseline risk factors, prior cancer therapies, and treatment with HMA-VEN rather than anthracycline exposure alone.

The occurrence of CV events has a substantial impact on patient care; in the present study, CV events led to an interruption of VEN in more than half (53%) of patients. Although not studied in AML, the negative impact of VEN interruption on survival has been demonstrated in chronic lymphocytic leukemia.^30^ The consistent association between CV events and mortality, which persisted even after the time-dependent propensity score matching, reinforces the prognostic importance of CV complications in this population.

### Strengths and limitations

This study has several strengths, including a large sample size, multicenter design, and time-dependent propensity score matching analysis, providing a robust assessment of the CV burden and its prognostic impact. This study was not designed to establish causal relationships or isolate the effect of HMA-VEN relative to other therapies, but rather to describe CV event patterns and their clinical implications within this population. Several limitations should be acknowledged. As a retrospective study, the analysis may be influenced by unmeasured factors not captured in the available data, as well as potential underreporting of CV events, particularly those not requiring hospitalization or formal cardiology evaluation. Biomarker data, including NT-proBNP, were unavailable for all patients, and the diagnosis of myocarditis was not uniformly confirmed by advanced imaging or biopsy. Additionally, while CV events were adjudicated, the heterogeneity in surveillance intensity across centers may have introduced detection bias. Disease status at HMA-VEN initiation (newly diagnosed vs relapsed/refractory AML) was not captured in a standardized manner across institutions, precluding a reliable stratified analysis. Furthermore, cycle-level VEN duration (eg, days of therapy during each cycle) was not uniformly documented across institutions and therefore could not be reliably abstracted. In the propensity score–matched analysis, the composite MACE definition included CV death, which may partially overlap with the mortality endpoint; however, CV death was infrequent in this cohort. Although several health systems included affiliated satellite sites and, in some cases, community hospitals, the study population may not fully reflect care delivered in smaller or independent community settings, where a substantial proportion of patients with AML are treated. These factors should be considered when interpreting the results and planning future prospective studies.

In summary, in this large multicenter retrospective study of patients with AML treated with HMA-VEN, CV complications were common, occurred early during treatment, and were associated with increased mortality and treatment disruption. Close CV monitoring and early intervention for cardiometabolic risk factors are important for managing CV events in this vulnerable population. Future prospective studies should validate these findings, explore mechanistic pathways further, and investigate preventive strategies to mitigate CV events in patients treated with VEN-HMA.

Perspectives **COMPETENCY IN MEDICAL KNOWLEDGE:** In patients with AML treated with HMA-VEN, CV complications seem to occur frequently and early, with a median onset of 120 days. The most common events include AF, LVEF decline, HF, and stroke/TIA. Diabetes and prior CAD are independently associated with higher risk, underscoring the importance of cardiometabolic risk assessment. Clinical vigilance is indicated; over half of patients who develop MACE require treatment interruption, which may compromise AML outcomes. During therapy, periodic CV surveillance may facilitate early detection of MACE, particularly in diabetic patients.

**TRANSLATIONAL OUTLOOK:** Prospective studies are needed to determine potential mechanisms of cardiovascular toxicity, refine risk stratification, and evaluate preventive strategies in patients receiving hypomethylating agents and venetoclax.

## Funding support and author disclosures

Dr Patel has received research funding (institutional) from 10.13039/100004319Pfizer, Sumitomo, 10.13039/100017655Incyte, and 10.13039/501100011725Servier; and has received honoraria from Jazz, AbbVie, Sobi, Astellas, Amgen, and Syndax. Dr Shallis has served in a consulting/advisory role and/or received honoraria from Bristol Myers Squibb, Gilead Sciences Inc, Kura Oncology, Rigel, Servier, Syndax Pharmaceuticals, and TScan Therapeutics, and has participated in a Steering Committee for Servier. Dr Tremblay receives contracted research funding paid to his institution from Sobi, Sumitomo, Cogent Biosciences, and Gilead and consulting fees from Sobi, AbbVie, Pharmaessentia, Geron, GSK, and Cogent Biosciences. All other authors have reported that they have no relationships relevant to the contents of this paper to disclose.
