# Supplementary material for: Cardiovascular Events in Patients With Acute Myeloid Leukemia Treated With Venetoclax: A Multicenter Cohort Study
Source: JACC Adv. 2026 Jun 17;5(6):102821. doi: 10.1016/j.jacadv.2026.102821 (PMC13308248; doi:10.1016/j.jacadv.2026.102821)
Supplement: Supplemental Material [file mmc1.pdf]

Supplemental Materials

Supplemental Table 1. Distribution of patients by participating centers

Center	Location	N (%)
Hospital of the University of Pennsylvania	Philadelphia, PA	195 (19.2)
Mount Sinai Hospital	New York, NY	89 (8.8)
Massachusetts General Hospital	Boston, MA	433 (42.8)
Yale New Haven Hospital	New Haven, CT	113 (11.2)
Northwestern Memorial Hospital	Chicago, IL	86 (8.5)
University of Chicago Medical Center	Chicago, IL	96 (9.5)
Total		1012 (100)

Supplemental Table 2. Detailed analysis of specific MACE in AML patients treated with HMA-VEN

**Decreased LVEF**	
De novo decrease in LVEF, n (%)	52 (5)
Time to decrease in LVEF, median [Q1-Q3]	102 [33 - 255]
Mean LVEF decrease, mean ± SD	19±9
NT-proBNP at baseline, median [Q1-Q3]	479 [99 - 4,444]
NT-proBNP at diagnosis, median [Q1-Q3]	6,940 [1,316 - 18,121]
Patients who recovered LVEF (>50%), n (%)	8 (15)
Patients who partially Recovered LVEF (increase, but <50%), n (%)	7 (13)
Patients with decreased LVEF who Died, n (%)	39 (75)
Management: Beta-Blockers, n (%)	17 (33)
Management: ACEI, n (%)	9 (17)
Management: MRA, n (%)	10 (19)
Management: SGLT2i, n (%)	6 (11)
Interruption of Venetoclax-HMA, n (%)	10 (19)
**Heart Failure**	
De Novo HF, n (%)	38 (4)
Time to HF, median [Q1-Q3]	128 [27 - 474]
LVEF at Baseline, mean ± SD	59±10
HFpEF, n (%)	13 (34)
NT-proBNP at Baseline, median [Q1-Q3]	896 [94 - 3,100]
NT-proBNP at Diagnosis, median [Q1-Q3]	3,562 [1,144 - 16,905]

Hemoglobin at Diagnosis, median [Q1-Q3]	8.2 [7 - 10]
CTCAE v5.0 HF Severity	
Grade 2, n (%)	17 (45)
Grade 3, n (%)	19 (50)
Grade 4, n (%)	2 (5)
Potential reason for HF: Takotsubo, n (%)	9 (24)
Potential reason for HF: Non-Ischemic Heart Disease, n (%)	7 (18)
Potential reason for HF: Chemotherapy Induced, n (%)	5 (13)
Potential reason for HF: Valvulopathy, n (%)	2 (5)
Potential reason for HF: Arrhythmic, n (%)	2 (5)
Management of HF: MRA, n (%)	8 (21)
Management of HF: ARNI, n (%)	6 (16)
Management of HF: SGLT2i, n (%)	4 (10)
Management of HF: Beta-Blockers, n (%)	13 (34)
Management of HF: ACEI, n (%)	4 (10)
Management of HF: Furosemide, n (%)	27 (71)
Interruption of Venetoclax-HMA, n (%)	6 (16)
**Angina**	
Angina, n (%)	26 (3)
Time to Angina, median [Q1-Q3]	212 [107 - 538]
Hemoglobin at Diagnosis, mean ± SD	8.4 (7 - 10)
Management: Beta-Blockers, n (%)	4 (15)
Management: ACEI, n (%)	2 (8)
Management: MRA, n (%)	2 (8)
Management: SGLT2i, n (%)	0
Management: ASA, n (%)	2 (8)
Management: Nitrate, n (%)	4 (15)
Management: CCB, n (%)	2 (8)
Interruption of Venetoclax-HMA, n (%)	2 (8)
**ACS**	
ACS, n (%)	23 (2)
Time to ACS, median [Q1-Q3]	121 [62 - 356]
Hemoglobin at Diagnosis, median [Q1-Q3]	8.4 [7 - 11]
Platelets at Diagnosis, median [Q1-Q3]	44 [18 - 183]
Management of ACS: Coronary Angioplasty, n (%)	8 (35)
Management of ACS: CABG, n (%)	0
Management of ACS: DAPT, n (%)	3 (13)
Management of ACS: Beta-Blockers, n (%)	8 (35)
Management of ACS: ACEI, n (%)	2 (9)
Management of ACS: Statin, n (%)	5 (22)
**VT/VF**	
De Novo VT/VF, n (%)	19 (2)
Time to VT/VF, median [Q1-Q3]	160 [34 - 574]
Management: External Electrical Cardioversion, n (%)	2 (10)
Management: ICD Shock, n (%)	2 (10)

Management: Beta-Blockers, n (%)	7 (37)
Management: Antiarrhythmics, n (%)	7 (37)
Management: Stopping of QT-prolonging Medications, n (%)	3 (16)
Interruption of Venetoclax-HMA, n (%)	2 (10)
**Myocarditis**	
Myocarditis, n (%)	9 (1)
Time to Myocarditis, median [Q1-Q3]	119 [65 - 300]
Diagnosis made by MRI, n (%)	6 (67)
Diagnosis made by Biopsy, n (%)	1 (11)
LVEF at Diagnosis, mean ± SD	50±20
NT pro-BNP at Baseline, median [Q1-Q3]	4,051 [443 - 7,521]
NT-pro-BNP at Diagnosis, median [Q1-Q3]	5,696 [1,346 - 12,188]

Values are median [interquartile range], n (%), or mean ± SD.

ACEI = angiotensin-converting enzyme inhibitor, ACS = acute coronary syndrome, ARNI = angiotensin receptor-neprilysin inhibitor, ASA = acetylsalicylic acid, CABG = coronary artery bypass graft, CCB = calcium channel blocker, DAPT = dual antiplatelet therapy, Echo = echocardiography, HF = heart failure, HFpEF = heart failure with preserved ejection fraction, ICD = implantable cardioverter defibrillator, ICOS = international cardio-oncology society, LVEF = left ventricular ejection fraction, MRA = mineralocorticoid receptor antagonist, MRI = magnetic resonance imaging, NT-proBNP = N-terminal pro-b-type natriuretic peptide, QT = QT interval on ECG, SGLT2i = sodium-glucose cotransporter-2 inhibitor, VT/VF = ventricular tachycardia/ventricular fibrillation

Supplemental Table 3. Univariable Fine-Gray Competing Risks Analysis of Variables Associated with Heart Failure and LVEF reduction

Variable	sHR	CI	P value
Age	0.99	0.98–1.01	0.45
Chronic Kidney Disease	0.68	0.41–1.11	0.12
History of Coronary Artery Disease	0.96	0.6–1.53	0.86
Diabetes	0.87	0.55–1.38	0.55
DNMT3A/TET2/ASXL1Mutation	0.84	0.57–1.24	0.37
Dyslipidemia	0.78	0.53–1.14	0.2
History of COPD	0.60	0.29–1.21	0.15
History of Heart Failure	0.93	0.52–1.67	0.81
History of Thoracic Radiation	0.92	0.44–1.9	0.82

Variable	sHR	CI	P value
Hypertension	0.86	0.59–1.27	0.45
IDH1/IDH2 Mutation	0.94	0.56–1.56	0.81
LVEF at Diagnosis	1.00	0.98–1.02	0.82
Number of Venetoclax Cycles Received	0.98	0.95–1.02	0.27
Obesity: BMI > 30 kg/m^2	0.75	0.47–1.2	0.23
Past Anthracyclines	1.10	0.70–1.72	0.69
Past Smoker	0.92	0.63–1.35	0.67
Previous History of Cancer	0.81	0.54–1.21	0.3
Previous SCT	0.87	0.57–1.35	0.54
Black/Hispanic ethnicity vs White	1.69	1.05–2.72	0.031
Sex	0.85	0.58–1.25	0.41
History of Stroke/TIA	0.11	0.02–0.78	0.027
COPD = chronic obstructive pulmonary disease, LVEF = left ventricular ejection fraction, TIA = transient ischemic attach, STC = stem cell transplant			

Supplemental Table 4. Multivariable Fine-Gray Competing Risks Analysis of Variables Associated with Heart Failure and LVEF Reduction

Variable	sHR	CI	P value
Age	1.0	0.98–1.01	0.960
History of Stroke/TIA	0.12	0.01–0.86	0.035
Black/Hispanic ethnicity vs White)	1.65	1.01–2.69	0.045
Sex	0.86	0.58–1.28	0.470
TIA = transient ischemic attack			

Supplemental Table 5. Univariable Fine-Gray Competing Risks Analysis of Variables Associated with Vascular Events

Variable	sHR	CI	P value
Age	1.02	1.00 - 1.04	0.015
History of Atrial Fibrillation	1.26	0.80 - 1.97	0.33
Azacytidine	1.23	0.79 - 1.93	0.36
BMI > 30 kg/m^2	1.58	1.05 - 2.38	0.028
History of Coronary Artery Disease	1.96	1.29 - 2.97	0.0017
Chronic Kidney Disease	1.48	0.97 - 2.28	0.069
History of COPD	1.45	0.85 - 2.46	0.17
Decitabine	0.95	0.62 - 1.44	0.80
Diabetes	2.11	1.41 - 3.15	<0.001
DNMT3A/TET2/ASXL1 Mutation	1.13	0.76 - 1.66	0.55
Dyslipidemia	1.55	1.04 - 2.31	0.030
History of Heart Failure	1.16	0.66 - 2.04	0.61
History of Thoracic Radiation	0.79	0.35 - 1.80	0.58
Hypertension	1.18	0.79 - 1.78	0.42
IDH1/IDH2 Mutation	1.05	0.65 - 1.70	0.85
LVEF at Diagnosis	1.01	0.98 - 1.03	0.70
Number of Venetoclax Cycles	0.99	0.96 - 1.02	0.54
Past Anthracyclines	1.13	0.55 - 2.32	0.73
Past Smoker	1.05	0.71 - 1.56	0.80
Previous History of Cancer	0.82	0.54 - 1.26	0.37
Previous SCT	0.70	0.44 - 1.10	0.12
Black/Hispanic ethnicity vs White)	1.72	0.99 - 2.98	0.054
Sex	1.14	0.77 - 1.70	0.52
Stroke/TIA	1.69	0.93 - 3.09	0.085
COPD = chronic obstructive pulmonary disease, LVEF = left ventricular ejection fraction, TIA = transient ischemic attach, STC = stem cell transplant			

Supplemental Table 6. Multivariable Fine-Gray Competing Risks Analysis of Variables Associated with Vascular Events

Variable	sHR	CI	P value
Age	1.01	0.99-1.04	0.083
BMI > 30 kg/m^2	1.46	0.95-2.26	0.084
History of Coronary Artery Disease	1.56	0.99-2.46	0.053
Diabetes	1.80	1.16-2.80	0.008
DNMT3A/TET2/ASXL1 Mutation	1.06	0.71-1.57	0.77
Dyslipidemia	1.16	0.76-1.76	0.49
IDH1/IDH2 Mutation	1.10	0.68-1.79	0.69
Black/Hispanic ethnicity vs White	1.30	1.00-1.69	0.05
Sex	0.99	0.66-1.50	0.99

Supplemental Table 7. Mortality Risk Factors in a Time-Dependent Matched Cohort of 645 Patients: A Multivariable Cox Regression Analysis

Variable	HR (95% CI)	P value
MACE	1.96 (1.59–2.42)	< 0.001
Age	1.01 (1.00–1.02)	0.006
Sex	0.87 (0.69–1.09)	0.23
Hypertension	0.96 (0.76–1.20)	0.72
Diabetes	1.08 (0.82–1.41)	0.59
Chronic kidney disease	1.05 (0.82–1.34)	0.70
History of Heart Failure	1.25 (0.96–1.62)	0.101
Previous Cancer Therapies	1.25 (1.02–1.54)	0.035
History of Coronary Artery Disease	1.35 (1.05–1.74)	0.020
**Death, n (%)**		**Time to death, median days [IQR]**
Matched cohort	397 (61)	289 [150 – 476]
MACE, n (%)	146 (73)	277 [107 – 524]

No MACE, n (%)	251 (56)	290 [165 – 466]
MACE = major cardiovascular events		

STROBE Statement—checklist of items that should be included in reports of observational studies

Item No

Recommendation

Response

Title and abstract

1

(*a*) Indicate the study's design with a commonly used term in the title or the abstract

Done (Title, Abstract)

(*b*) Provide in the abstract an informative and balanced summary of what was done and what was found

Done (Abstract, page 3-4)

Introduction

Background/rationale

2

Explain the scientific background and rationale for the investigation being reported

Done (Introduction, Page 4-5)

Objectives

3

State specific objectives, including any prespecified hypotheses

Done (Introduction, Page 5)

Methods

Study design

4

Present key elements of study design early in the paper

Done (Methods, Page 5)

Setting

5

Describe the setting, locations, and relevant dates, including periods of recruitment, exposure, follow-up, and data collection

Done (Methods, Page 5)

Participants

6

(*a*) *Cohort study*—Give the eligibility criteria, and the sources and methods of selection of participants. Describe methods of follow-up
Case-control study—Give the eligibility criteria, and the sources and methods of case ascertainment and control selection. Give the rationale for the choice of cases and controls
Cross-sectional study—Give the eligibility criteria, and the sources and methods of selection of participants
Done (Methods Page 5)

(*b*) *Cohort study*—For matched studies, give matching criteria and number of exposed and unexposed
Case-control study—For matched studies, give matching criteria and the number of controls per case
Done (Methods Page 7)

Variables

7

Clearly define all outcomes, exposures, predictors, potential confounders, and effect modifiers. Give diagnostic criteria, if applicable
Done (Methods Page 5)

Data sources/ measurement

8*

For each variable of interest, give sources of data and details of methods of assessment (measurement). Describe comparability of assessment methods if there is more than one group
Done (Methods Page 5)

Bias

9

Describe any efforts to address potential sources of bias
Done (Methods Page 5)

Study size

10

Explain how the study size was arrived at
Done (Methods Page 5)

Quantitative variables

11

Explain how quantitative variables were handled in the analyses. If applicable, describe which groupings were chosen and why
Done (Methods Page 5)

Statistical methods

12

(*a*) Describe all statistical methods, including those used to control for confounding
Done (Methods Page 7)

(*b*) Describe any methods used to examine subgroups and interactions
Done (Methods Page 7)

(*c*) Explain how missing data were addressed
Done (Table 1)

(*d*) *Cohort study*—If applicable, explain how loss to follow-up was addressed
Case-control study—If applicable, explain how matching of cases and controls was addressed
Cross-sectional study—If applicable, describe analytical methods taking account of sampling strategy
Done (Methods Page 7)

(*e*) Describe any sensitivity analyses
Done (Methods Page 8)

Continued on next page

Results

Participants

13*

(a) Report numbers of individuals at each stage of study—eg numbers potentially eligible, examined for eligibility, confirmed eligible, included in the study, completing follow-up, and analysed

N/A

(b) Give reasons for non-participation at each stage

N/A

(c) Consider use of a flow diagram

N/A

Descriptive data

14*

(a) Give characteristics of study participants (eg demographic, clinical, social) and information on exposures and potential confounders

Done (Results, Page 9, Table 1)

(b) Indicate number of participants with missing data for each variable of interest

Done (Table 1)

(c) *Cohort study*—Summarise follow-up time (eg, average and total amount)

Done (Results, Page 9)

Outcome data

15*

Cohort study—Report numbers of outcome events or summary measures over time

Done (Results, Page 9, Table 2)

Case-control study—Report numbers in each exposure category, or summary measures of exposure

N/A

Cross-sectional study—Report numbers of outcome events or summary measures

N/A

Main results

16

(*a*) Give unadjusted estimates and, if applicable, confounder-adjusted estimates and their precision (eg, 95% confidence interval). Make clear which confounders were adjusted for and why they were included

Done (Results, Page 9-12)

(*b*) Report category boundaries when continuous variables were categorized

N/A

(*c*) If relevant, consider translating estimates of relative risk into absolute risk for a meaningful time period

N/A

Other analyses

17

Report other analyses done—eg analyses of subgroups and interactions, and sensitivity analyses

Done (Results, Page 9-12, supplemental Tables)

Discussion

Key results

18

Summarise key results with reference to study objectives

Done (Discussion, Page 12)

Limitations

19

Discuss limitations of the study, taking into account sources of potential bias or imprecision. Discuss both direction and magnitude of any potential bias

Done (Discussion, Page 16)

Interpretation

20

Give a cautious overall interpretation of results considering objectives, limitations, multiplicity of analyses, results from similar studies, and other relevant evidence

Done (Discussion, Page 12-16)

Generalisability

21

Discuss the generalisability (external validity) of the study results

Done (Discussion, Page 16)

Other information

Funding

22

Give the source of funding and the role of the funders for the present study and, if applicable, for the original study on which the present article is based

Done (Title Page, Page 1)

*Give information separately for cases and controls in case-control studies and, if applicable, for exposed and unexposed groups in cohort and cross-sectional studies.

Note: An Explanation and Elaboration article discusses each checklist item and gives methodological background and published examples of transparent reporting. The STROBE checklist is best used in conjunction with this article (freely available on the Web sites of PLoS Medicine at http://www.plosmedicine.org/, Annals of Internal Medicine at http://www.annals.org/, and Epidemiology at http://www.epidem.com/). Information on the STROBE Initiative is available at www.strobe-statement.org.
